# Opening of pannexin- and connexin-based channels increases the excitability of nodose ganglion sensory neurons

**DOI:** 10.3389/fncel.2014.00158

**Published:** 2014-06-20

**Authors:** Mauricio A. Retamal, Julio Alcayaga, Christian A. Verdugo, Geert Bultynck, Luc Leybaert, Pablo J. Sáez, Ricardo Fernández, Luis E. León, Juan C. Sáez

**Affiliations:** ^1^Facultad de Medicina, Centro de Fisiología Celular e Integrativa, Clínica Alemana Universidad del DesarrolloSantiago, Chile; ^2^Departamento de Fisiología, Facultad de Ciencias Biológicas, Pontificia Universidad Católica de ChileSantiago, Chile; ^3^Laboratorio de Fisiología Celular, Departamento de Biología, Facultad de Ciencias, Universidad de ChileSantiago, Chile; ^4^KU Leuven, Laboratory of Molecular and Cellular Signaling, Department of Cellular and Molecular MedicineLeuven, Belgium; ^5^Physiology Group, Department of Basic Medical Sciences, Faculty of Medicine and Health Sciences, Ghent UniversityGhent, Belgium; ^6^Facultad de Ciencias Biológicas y Facultad de Medicina, Universidad Andrés BelloSantiago, Chile; ^7^Facultad de Medicina, Centro de Genética Humana, Clínica Alemana Universidad del DesarrolloSantiago, Chile; ^8^Centro Interdisciplinario de Neurociencias de Valparaíso, Instituto MilenioValparaíso, Chile

**Keywords:** glial satellite cells, connexon, peripheral glial cells, sensory ganglia, nodose ganglia

## Abstract

Satellite glial cells (SGCs) are the main glia in sensory ganglia. They surround neuronal bodies and form a cap that prevents the formation of chemical or electrical synapses between neighboring neurons. SGCs have been suggested to establish bidirectional paracrine communication with sensory neurons. However, the molecular mechanism involved in this cellular communication is unknown. In the central nervous system (CNS), astrocytes present connexin43 (Cx43) hemichannels and pannexin1 (Panx1) channels, and the opening of these channels allows the release of signal molecules, such as ATP and glutamate. We propose that these channels could play a role in glia-neuron communication in sensory ganglia. Therefore, we studied the expression and function of Cx43 and Panx1 in rat and mouse nodose-petrosal-jugular complexes (NPJcs) using confocal immunofluorescence, molecular and electrophysiological techniques. Cx43 and Panx1 were detected in SGCs and in sensory neurons, respectively. In the rat and mouse, the electrical activity of vagal nerve increased significantly after nodose neurons were exposed to a Ca^2+^/Mg^2+^-free solution, a condition that increases the open probability of Cx hemichannels. This response was partially mimicked by a cell-permeable peptide corresponding to the last 10 amino acids of Cx43 (TAT-Cx43CT). Enhanced neuronal activity was reduced by Cx hemichannel, Panx1 channel and P2X_7_ receptor blockers. Moreover, the role of Panx1 was confirmed in NPJc, because in those from Panx1 knockout mice showed a reduced increase of neuronal activity induced by Ca^2+^/Mg^2+^-free extracellular conditions. The data suggest that Cx hemichannels and Panx channels serve as paracrine communication pathways between SGCs and neurons by modulating the excitability of sensory neurons.

## Introduction

Plasma membrane hemichannels are composed of six protein subunits named connexins (Cxs). When two hemichannels from apposing cells contact, a gap junction channel (GJC) can be formed. GJCs communicate the cytoplasm of neighboring cells, whereas hemichannels allow the exchange of ions and small molecules between intra- and extracellular media. In addition, hemichannel opening is associated with the release of signaling molecules such as ATP, NAD^+^, glutamate and prostaglandin-E_2_ (Bruzzone et al., 2001; Stout et al., 2002; Ye et al., 2003; Cherian et al., 2005). On the other hand, pannexins (Panxs) are membrane proteins that also form channels at the plasma membrane (Bruzzone et al., 2003) and that share some properties with Cx hemichannels. For example, Panx1 channels are permeable to large molecules such as calcein and HEPES (Thompson et al., 2006; Romanov et al., 2012a,b). However, the permeability of Panx1 channels to ATP remains a matter of controversy, mainly because (i) Panx1 channels seem to be impermeable to ATP (Romanov et al., 2012a,b) and (ii) a high extracellular ATP concentration acts as blocker of Panx channels (Qiu and Dahl, 2009).

In the central nervous system (CNS), the expression and function of channels formed by Cxs or by Panxs have received particular attention (Sáez et al., 2003; Nagy et al., 2004; Theis et al., 2005; Thompson and Macvicar, 2008). However, in the peripheral nervous system (PNS), these channels have been less studied (Pannese, 2010). In the PNS, cell bodies of sensory neurons are located in sensory ganglia. These neurons have a pseudomonopolar shape and each perikaryon is fully surrounded by a cellular layer of glia, called satellite glial cells (SGCs). It has been proposed that these SGCs separate each neuronal body from one another, precluding the establishment of any type of synapse (Pannese, 1981, 2010). Consistent with this proposal, numerous ultrastructural and dye coupling studies have failed to find morphological or functional evidence of GJCs between sensory neurons or between sensory neurons and SGCs (Stensaas and Fidone, 1977; Shinder et al., 1998; Sakuma et al., 2001; Zuriel and Devor, 2001; Chen et al., 2002; Hanani et al., 2002; Pannese et al., 2003; Huang et al., 2006). In contrast, SGCs are coupled electrically and metabolically through GJCs (Sakuma et al., 2001; Huang et al., 2006; Pannese, 2010). It is known that SGCs influence neuronal excitability (Hanani, 2005; Huang and Hanani, 2005) by modifying the extracellular K^+^ concentration and the intracellular free Ca^2+^ concentration (Pannese, 1981; Suadicani et al., 2009). Additionally, SGCs can modify neuronal responses to neurotransmitters (Pannese, 1981; Mandelzys and Cooper, 1992; Heblich et al., 2001).

The molecular mechanism of the autocrine/paracrine communication between SGCs and neurons is not fully understood. Hence, in the present work we tested whether cells from visceral sensory ganglia present functional Cx- and/or Panx- based channels in their plasma membranes. The mRNAs and proteins of Cx43 and Panx1 were detected in the nodosal petrosal jugular complex (NPJc) by RT-PCR, Western blot and confocal immunofluorescence. Electrophysiological studies *in vitro* showed hemichannels opening in response to a Ca^2+^/Mg^2+^-free solution (mHBSS), which is associated with increased electrical activity of nodose neurons. Compared with NPJc of wild type mice, ganglia from Panx1 knockout mice exposed to Ca^2+^/Mg^2+^-free solution showed a decreased response. Similar results were obtained when the P2X_7_ receptors were pharmacologically inhibited. Thus, we postulate that Cx hemichannels and Panx channels serve as paracrine communication pathways in sensory ganglia, determining the electrical excitability of these PNS neurons.

## Materials and methods

### Chemicals

Fluoromount-G was purchased from Electron Microscopy Science (Ft. Washington, PA, USA). Distilled water, collagenase type A, deoxyribonuclease I, poly-D-lysine, 18β-glycyrrhetinic acid (βGA), 2′(3′)-O-(4-benzoylbenzoyl) adenosine 5′-triphosphate triethylammonium salt (BzATP), periodate oxidized adenosine 5′-triphosphate (oATP), acetyl choline and Probenecid were obtained from Sigma-Aldrich (St. Louis, MO, USA). Mouse nerve growth factor (NGF 7S) was obtained from Invitrogen (Carlsbad, CA, USA). Gap27 peptide was obtained from AnaSpec (Fremont, CA, USA). Mouse monoclonal glial glutamine synthetase (GS) antibody was obtained from Santa Cruz Biotechnology. Previously described rabbit polyclonal anti-Cx43 (see Brañes et al., 2002) and rabbit polyclonal anti-Panx1 (see Riquelme et al., 2013) sera were used.

### Animals

Male Sprague-Dawley rats and male and female C57BL/6 mice were obtained from the animal research facilities of the Faculty of Biological Sciences of the Pontificia Universidad Católica de Chile. Panx1 knock-out (KO) C57BL/6 mice previously described by Bargiotas et al. (2011) were kindly provided by Dr. Hannah Monyer, University Heidelberg, Germany. These animals were bred in the animal research facilities of the Pontifícia Universidad Católica de Chile. Wild type C57BL/6 mice were used as controls. The use of KO mice was limited to crucial experiments to reduce the number of animals sacrificed.

The Commission of Bioethics and Biosafety from our respective universities approved all experimental protocols, which were performed according to the “Guide for the Care and Use of Laboratory Animals,” Institute of Laboratory Animal Research Commission on Life Sciences, National Research Council (National Academy Press, Washington, DC 1996).

### Ganglion extraction

NPJc were obtained from 6-8-week-old Sprague-Dawley rats and from C57BL/6 mice (wild type and Panx1 knock out). Rats and mice of both sexes were anesthetized with sodium pentobarbitone 60 mg/kg which was administered intraperitoneally (i.p.) and supplemented with additional doses when necessary to maintain a light level of surgical anesthesia (Stage 3, plane 2). The neck was opened through a midline incision. Then, the vagus nerve was dissected, and its peripheral processes were cut ~1 centimeter distal to the ganglion. Next, each NPJc was exposed and its central process was cut approximately 1 mm from its apparent central border. After both NPJc were removed, the animals were euthanized by an overdose (180 mg/kg) of pentobarbitone.

### Immunoblot

Ganglia were dissected as indicated above and then placed in ice cold phosphate buffered saline solution (PBS) containing 200 μg/mL soybean trypsin inhibitor, 1 mg/mL benzamidine, 1 mg/mL ε-aminocaproic acid and 2 mM phenylmethylsulfonyl fluoride and phosphatase inhibitors (20 mM Na_4_P_2_O_7_ and 100 mM NaF). Then, ganglia were cut in small pieces with thin scissors and lysed by sonication. Samples were resuspended in Laemmli buffer and stored at −80°C, or proteins were resolved immediately in 8% SDS-PAGE. After electrophoresis, proteins were electrotransferred to VDF membranes incubated in PBS-BLOTTO (5% non-fat milk in PBS) for 45 min to block non-specific binding sites. Then, blots were incubated with primary antibodies for 1 h at room temperature, followed by several washes in PBS, and then incubated with HRP-conjugated goat anti-rabbit IgGs (secondary antibodies) for 1 h at room temperature. An ECL SuperSignal kit was used according to the manufacturer's instructions to detect immunoreactivity.

### RT-PCR procedure

NPJc from male Sprague-Dawley rats anesthetized with pentobarbitone (60 mg/kg i.p.) were excised as described above and immediately transferred into cold modified (Ca^2+^/Mg^2+^-free) Hanks' balanced salt solution (mHBSS). Due to the small size of NPJc, they were pooled from 4 rats (8 NPJc) and stored in TRIzol-reagent for RNA extraction (For details, see Fernández et al., 2011). Briefly, RNA was prepared by the acid guanidinium-phenol-chloroform method, using TRIzol reagent (Invitrogen, Carlsbad, CA, USA) according to the manufacturer's instructions. Tissues were homogenized on ice. Then, quantification and purity checks of total RNA were performed spectrophotometrically and electrophoretically, respectively. After DNase treatment, total RNA (2 μg) was reverse transcribed into single strand cDNA using random primers and Moloney murine leukemia virus reverse transcriptase (M-MLV RT, 200 U/μL, Invitrogen, Carlsbad, CA. USA). Primer pairs used to amplify the coding regions for Cxs and for Panxs are listed in Table 1. Total RNA from rat brains was processed as mentioned above and used as positive control. As negative controls, reactions were also performed using samples without RNA or with cDNA prepared in the absence of RT enzyme. The amplified products were separated on a 2% agarose gel, which was subsequently stained with ethidium bromide (Sigma-Aldrich) and photographed under UV illumination. Images were taken with a digital camera.

**Table 1:** **Sequences of RT-PCR primers used to detect Cxs and Panxs mRNAs in rat's NPJcs**.

**Connexin**	**Sequence 5′–3′**
Cx26F	ACGTTGGCCTTTTGGTTATG
Cx26R	TGTTGCGGGCTGTACTCAG
Cx37F	TTCTGGCCACCCTGGGGGGC
Cx37R	GGCTGGACCATGGAGCCGGT
^*^Cx43F	TACCACGCCACCACTGGCCCA
^*^Cx43R	ATTCTGGTTGTCGTCGGGGAAATC
Cx45F	GCAGAACAAAGCCAATATCGCCCA
Cx45R	TTCTGGTGATGGTAGGCCTGGATT
**Pannexin**	**Sequence 5′–3′**
^*^Panx1F	AGAGCTAGCTTTGTTCCCGG
^*^Panx1R	AGCTTATCTGGGTACCGATGG A
Panx2F	TGGACATCGTATTGCTCTGC
Panx2R	CCACGTTGTCGTACATGAGG

### Electrophysiology

NPJc were placed in ice-chilled Hanks' balanced salt solution (HBSS), and the connective tissue over the ganglia was carefully removed. The NPJc were transferred into a two-compartment chamber kept at 38.0 ± 0.5°C and superfused with HBSS supplemented with 5 mM HEPES buffer, pH 7.43, which was equlibrated with air and flowed at approximately 1.2 mL/min. Ganglia were placed in the 0.2 mL capacity lower compartment, over a pair of platinum electrodes, and pinned to the bottom of the chamber. The electrodes were connected to a stimulator, and a thermistor was in the superfusion channel near the ganglion surface. The vagus nerve (VN) was placed on paired Pt recording electrodes and lifted into the upper compartment of the chamber which was filled with mineral oil. Recording electrodes were connected to an AC-preamplifier (Model 1800; A-M Systems, USA) and the resulting electroneurogram was amplified, displayed on an oscilloscope, and recorded on video cassette tapes. The electroneurogram was also fed to a spike amplitude discriminator whose standarized pulses were counted at 1-s intervals to assess the frequency of discharge (*f_vn_*), which was also digitized online through an AD board displayed on a computer using custom software and saved as ASCII encoded text files for later analysis. Drugs were applied in 10–50 μL boluses by micropipettes whose tips were placed approximately 1 mm distance from the exposed surface of the NPJc. All peptides were applied in a 50 μl bolus at a final concentration of 100 μM in a stop flow configuration for a maximum of 15 min. The mean basal activity (bas *f_VN_*) was computed in the 30-s period prior before any experimental procedure. The steady-state frequency was computed in a 30-s interval at the end of an experimental superfusion period. The initial increase in frequency was computed in a 2 min interval after 1 min of an experimental superfusion period. The discharge frequency rate of change (Δ*f*/Δ*t*) was computed in 3-s intervals for each frequency point (1-s). The maximal values for the onset and end of the responses were used to compute the mean absolute value of Δ*f*/Δ*t* (|Δ*f*/Δ*t*|).

### Confocal microscopy analysis

Rat NPJc were embedded in a resin (OCT) to allow sectioning of frozen tissue, frozen in liquid nitrogen and stored at −80°C. Sagittal cryostat sections (10 μm thick) were prepared fixed with 4% paraformaldehyde for 20 min at room temperature, washed three times with PBS and stored at 4°C. A blocking solution, which contained 1% IgG-free BSA, 50 mM NH_4_Cl and 0.05% Triton X-100 in PBS was used to permeabilize and to block unspecific reactive sites. Panx1 and Cx43 were detected with rabbit polyclonal anti-Panx1 and anti-Cx43 sera, respectively. These antibodies were properly diluted in blocking solution and incubated overnight. After washing with PBS, Cy2 conjugated goat anti-rabbit (1:300) IgGF(ab') fragments were incubated for 45 min at room temperature to detect bound primary antibody. After Cy2 washing, a monoclonal anti-glutamine synthetase (GS) antibody was added to samples for 3 h at proper dilution. Then, samples were washed and incubated with a Cy3 conjugated anti-mouse IgGF(ab') and fragments were incubated as described previously for Cy2. DAPI Fluoromount-G (Electron Microscopy Sciences, Washington, PA) was used as an anti-fade solution to mount samples. Images were examined using a spectral two-photon confocal laser scanning microscope (Zeiss, Spectral Confocal Microscope, LSM780). Images with an optical thickness of 0.3 μm were obtained using a Plan-Apochromat 63x/1.40 Oil DIC M27 objective and then analyzed using Carl Zeiss image analysis software (Zen 2011).

### Statistical analyses

Results are presented as the mean ± standard error (SE). Two populations were compared using Student's *t*-test or a nonparametric test, according to the data structure. Multiple populations were analyzed using repeated measures two-way ANOVA with multiple comparisons post-hoc tests. Analyses were performed using GraphPad Prism 6.03 (GraphPad Software, La Jolla, CA, USA) or Microsoft Excel programs. *P* < 0.05 was considered statistically significant. All comparisons of experimental data were performed with two-tailed tests, whereas the comparison of indexes was performed with one-tail statistics.

## Results

### Expression and cellular distribution of Cx43 and Panx1 in NPJc

**Figure 1:**
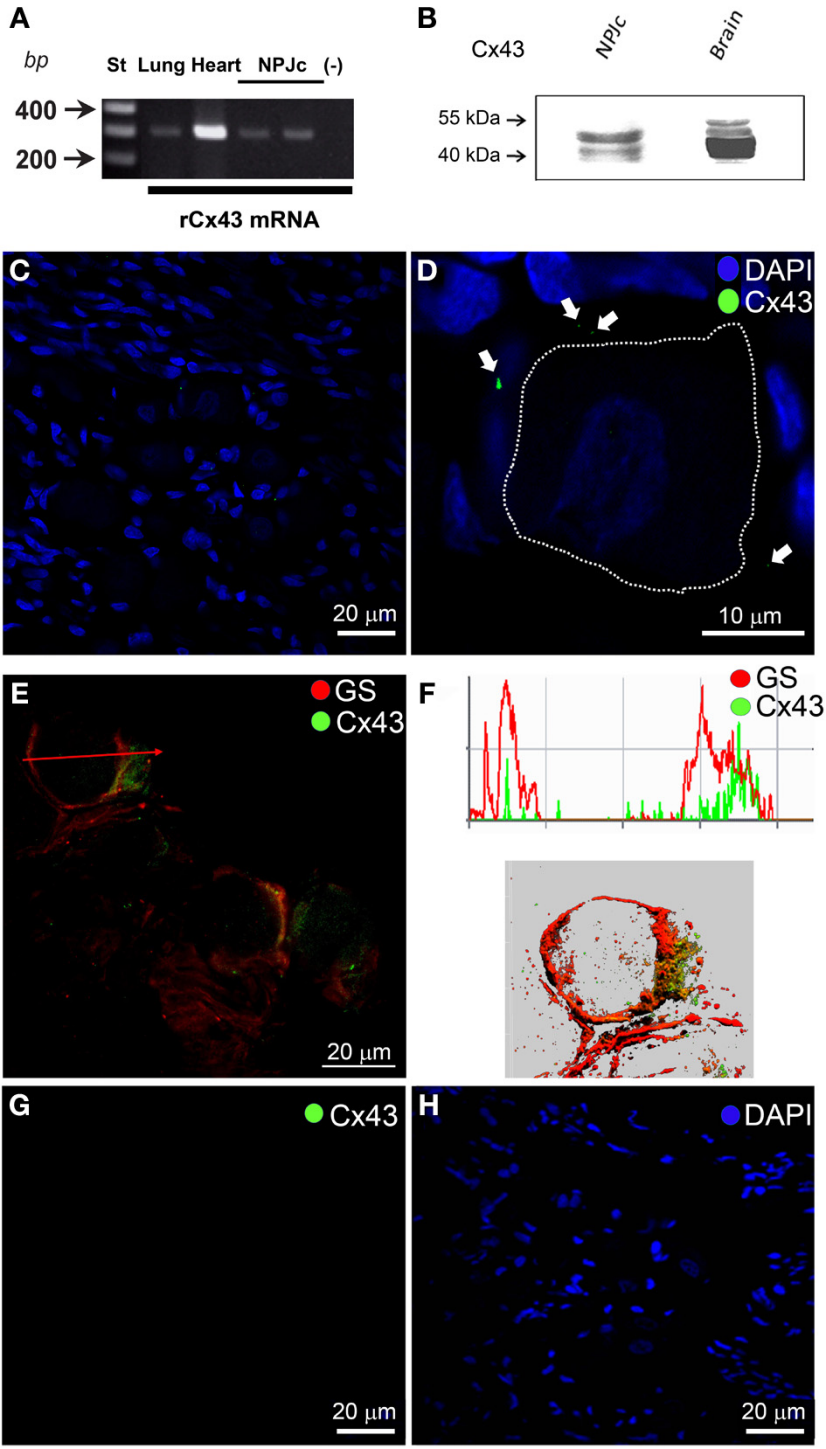
**Cx43 is expressed in NPJcs**. **(A)** RT-PCR detection of Cx43 mRNA in total extracts of rat NPJcs. Each lane shows Etd^+^-stained amplicons: lanes 1 and 2, lung and heart used as positive controls, lanes 3 and 4 two independent NPJc samples and lane 5 a reaction performed without RNA (negative control) (*n* = 4). **(B)** Levels of Cx43 in total NPJc homogenates were analyzed by Western blot. Cx43 was detected in total homogenates of NPJc and brain (50 μg of protein). **(C)** Indirect immunofluorescence of Cx43 in a slice of NPJc. **(D)** Digital zoom of a single neuron soma, where white arrows indicate the presence of Cx43 and the neuronal soma was drawn with a white line. **(E)** Co-localization of Cx43 (green) and GS (red) was evident in a zone (NPJc edge) where both neuronal bodies and SGC are present. **(F)** Digital analyses of the fluorescence observed in **(E)** (Red arrow), and the Z-reconstruction of this fluorescent signal. **(G)** Indirect immunofluorescence in a slice of NPJc when Cx43 primary antibody was omitted. **(H)** DAPI staining shows cell nucleus of same slice as in panel **(G)**.

The presence and distribution of Cx43 and Panx1 were studied in NPJc by RT-PCR, Western blot and confocal immunofluorescence analyses. In NPJc extracts, Cx43 mRNA was detected in each preparation analyzed (Figure 1A). We used extracts of lung and heart as positive controls (Figure 1A) and an enzyme mix without RNA as the negative control [Figure 1A, (-)]. In Western blot analyses, Cx43 was detected in NPJc samples (Figure 1B, line NPJc) and in rat brain extracts which were used as positive control. In both cases, several bands with electrophoretic mobility of approximately 43 kDa were observed. We suggest that in both samples, Cx43 present different degrees of phosphorylation, which is reflected as bands with different electrophoretic mobility (Kadle et al., 1991).

In rat NPJc sections, Cx43 reactivity was extremely weak and distributed as small dots all over the ganglion cross section (Figure 1C, nuclei were stained with DAPI). By zooming in an area containing a single neuron, Cx43 immunoreactivity was clearly observed not in the neuronal body but surrounding the neural body (Figure 1D, white arrows). To confirm that Cx43 was localized in SGCs, we performed double immunostaining with glutamine synthetase (GS), which is a SGC marker (Jasmin et al., 2010). Higher reactivity of Cx43 was usually detected in small zones of the ganglion periphery. In this case, a clear co-immunolabeling between GS and Cx43 was observed (Figure 1E). The digital analysis of the fluorescence signals obtained in Figure 1E (Red arrow) confirmed that Cx43 expression (Figure 1F, green line) was strongly associated with presence of GS (Figure 1F, red line). To clarify these findings, we used these data to generate a Z-plane rendered reconstruction (Figure 1F, lower panel). When Cx43 primary antibody was omitted, no immunolabeling was observed (Figures 1G,H).

**Figure 2:**
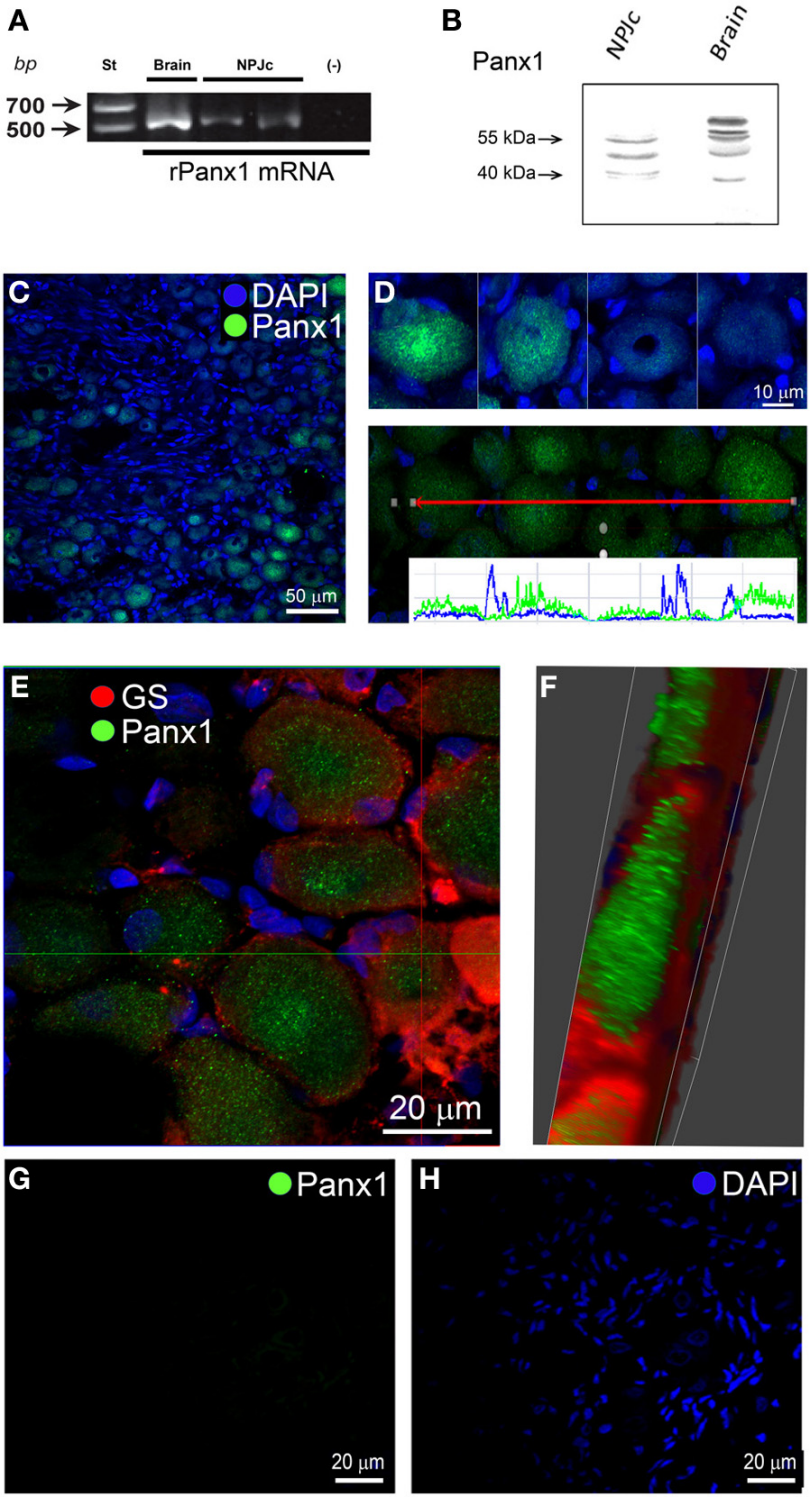
**Panx1 is expressed in NPJcs. (A)** RT-PCR detection of Panx1 mRNA in total extracts of NPJcs. Each lane shows Etd^+^-stained amplicons: lane 1 correspond to brain extracts used as positive control; lanes 2 and 3 correspond to two independent NPJc samples and lane 4 correspond to a reaction that was performed without RNA (negative control). **(B)** Panx1 protein was detected by Western blot analyses. In NPJc homogenates, several reactive bands with electrophoretic mobility were found. However, a band of approximately 50 kDa was clearly detected in rat NPJc and brain samples. **(C)** Confocal immunofluorescence detection of Panx1 in NPJc slices. **(D)** Four examples of neurons expressing Panx1 are presented (upper panel). Digital analysis of a zone of the NPJc expressing Panx1 (lower panel, green line) and DAPI staining (lower panel, blue line) were performed, showing that Panx1 does not co-localize with DAPI, which mainly marks the SGC nuclei. **(E)** Co-immunolabeling of Panx1 (Green) and GS (Red) **(F)** 3D module of ZEN 2011 used to visualize the data by transparent rendering Z-reconstruction. **(G)** Indirect immunofluorescence in a slice of NPJc when Panx1 primary antibody was omitted. **(H)** DAPI staining showing cell nuclei of the same field presented in panel **(G)**.

Panx1 mRNA was also detected in NPJc extracts (Figure 2A). Samples from rat brain were used as positive control. In Western blot analyses, a band with electrophoretic mobility of approximately 50 kDa was evident in NPJc extracts. Other bands with higher and lower electrophoretic mobility were also detected (Figure 2B, NPJc). In rat brain samples, an approximately 50 kDa band was also detected; however, bands with less electrophoretic mobility were also evident (Figure 2B, Brain). These bands could be the result of post-translational modification, such as glycosylation (Boassa et al., 2007). Similar results have been obtained in samples from rat brain using different Panx1 antibodies (Cone et al., 2013). We analyzed the localization of Panx1 in NPJc and found that Panx1 reactivity was localized primarily as dotted marks in the cytoplasm of sensory neurons (Figure 2C). Panx1 was located mostly in neurons with variable degrees of reactivity (Figure 2D, upper panel). Analyses of DAPI and Panx1 localization (Figure 2D, lower panel) revealed that Panx1 (green line) is excluded from zones with strong DAPI fluorescence, corresponding mostly to SGC nuclei (blue line). This finding suggests that Panx1 is expressed, mainly, if not exclusively, in sensory neurons. To confirm this finding, double immunostaining of Panx1 and GS was performed, which showed Panx1 localization in the soma of sensory neurons (Figure 2E), and not in GS reactive zones. Rendering of a single neuron confirmed that Panx1 is localized in neurons (green) and not in surrounding SGCs (red) (Figure 2F). When Panx1 primary antibody was omitted, no immunolabeling was observed (Figures 2G,H).

### Cx43 hemichannel opening increases nodosal neuronal activity

**Figure 3:**
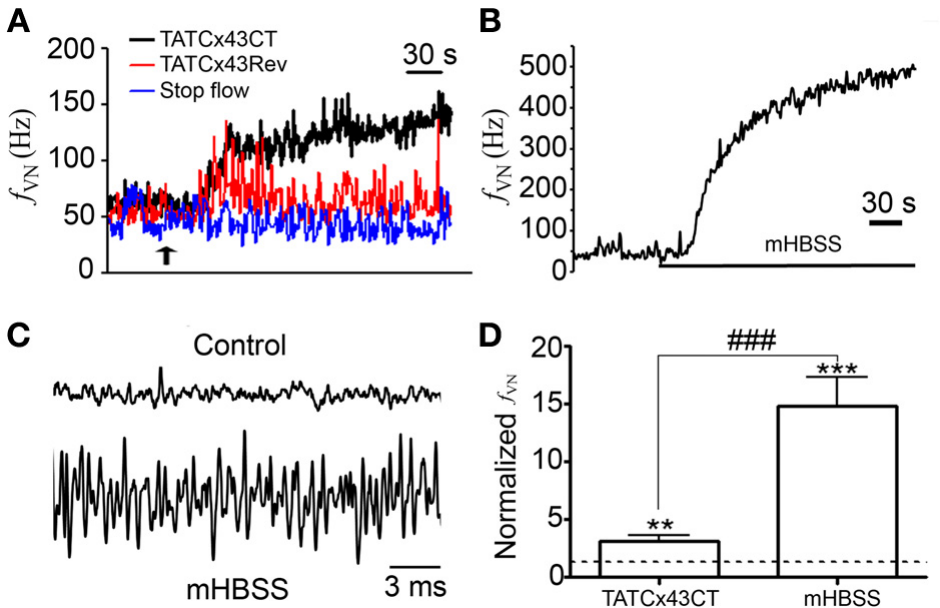
**TATCx43CT and mHBSS enhance the neuronal activity of rat NPJcs**. Rat NPJc were placed in a recoding chamber superfused with Hanks' solution (HBSS) and the frequency of discharge was measured in the vagus nerve (*f_VN_*). **(A)** Effect of (10 μL) (black arrow) 100 μM TATCx43CT (black trace) or 100 μM TATCx43Rev (red trace) applied to NPJcs *ex vivo* on *f_VN_*. Stop-flow technique did not modify the basal frequency of discharge (blue trace). **(B)** *f_VN_* recorded before and during (indicated by continuous line) superfusion with mHBSS. **(C)** Spontaneous activity recorded under control conditions (upper trace) and during superfusion with Ca^2+^/Mg^2+^-free HBSS (mHBSS; lower trace). **(D)** Graph showing the normalized effect of TATCx43CT (*n* = 9) and mHBSS (*n* = 21) over the electrical activity of NPJcs compared with control conditions (dashed line). Statistical significances; ^**^P < 0.01 and ^***^P < 0.001 different compared with the basal (pre stimulus) condition, ^###^*P* < 0.001 between stimuli. Wilcoxon matched-pairs signed rank test.

We demonstrated that NPJc cells express at least Cx43. The opening of channels formed by these proteins is known to permit the release of several neurotransmitters (Sáez et al., 2003). TATCx43CT is a peptide corresponding to the last 10 amino acids of the C-terminal tail of Cx43 which favors the opening of Cx43 hemichannels by preventing their closure at high cytoplasmic Ca^2+^ concentration (Ponsaerts et al., 2010; De Bock et al., 2012; Iyyathurai et al., 2013). We explored whether this peptide could increase the electrical activity of sensory neurons projecting through the vagus nerve in the stop-flow mode. Stop-flow *per se* (for a maximum of 15 min) did not affect neuronal basal discharges (Figure 3A, blue line). The application of 100 μM TATCx43CT to rat NPJc *in vitro* induced a fast and sustained increase in the nodose neuronal discharge frequency recorded in the vagus nerve (*f_VN_*) (Figure 3A, black line). This response was maintained for at least 15 min. The application of 100 μM TATCx43Rev [a peptide that has the same sequence as TATCx43CT but in a reversed order and that does not interact with Cx43 hemichannels (Ponsaerts et al., 2010)] only induced a small and transient (30–90 s) increase in neuronal activity (Figure 3A, red line). This finding suggests that Cx43 hemichannel opening increases the neuronal discharge of action potentials.

**Figure 4:**
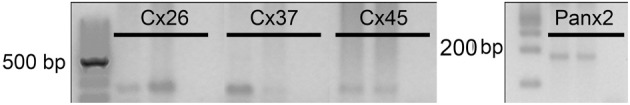
**Presence of Cxs 26, 37, 45, and Panx2 in NPJcs**. mRNAs of Cxs 26, 37 and 45 and Panx2 were detected in total RNA extracted from rat NPJcs and in RT-PCR analyses. The primers used are listed in Table 1.

A method to induce Cx43 hemichannel opening is to expose cells to an extracellular solution without divalent cations (Sáez et al., 2010). Our previous results supported the idea that Cx43 hemichannels are present in NPJc cells. Thus, we tested the effect of Hanks' solution with nominal zero Ca^2+^ and Mg^2+^ (mHBSS) on neuronal activity. In rat NPJc superfused with mHBSS, an increase of the basal frequency of discharge was recorded (Figure 3B). This increase in neuronal activity began 0.5–3 min after changing regular Hanks' solution (HBSS) to mHBSS and persisted for at least 30 min. According to these results, few spontaneous action potentials were detected in control conditions (Figure 3C, upper recording), however, during mHBSS or after TATCx43CT addition, spontaneous action potentials were frequently recorded (Figure 3C, lower recording). The quantification of these results shows that mHBSS increased the neuronal activity 14.8 ± 2.4 times (*n* = 21), which was 4.2 times larger than that of TATCx43CT (3.1 ± 0.5 times; *n* = 7) (Figure 3D, *P* < 0.001). This result can be explained by the incomplete stimulation of Cx43 hemichannel opening by TATCx43CT, and by the presence of hemichannels composed of other Cxs. We analyzed the presence of mRNAs of other Cxs and Panxs present in the nervous system (Nagy et al., 2004; Thompson and Macvicar, 2008). The mRNAs for Cx26, Cx37, Cx45, and Panx2 were detected (Figure 4), showing that sensory neurons and/or SGCs express several Cx isoforms and at least one other Panx isoform.

### Increased neuronal activity induced by mHBSS is sensitive to Cx hemichannel blockers

**Figure 5:**
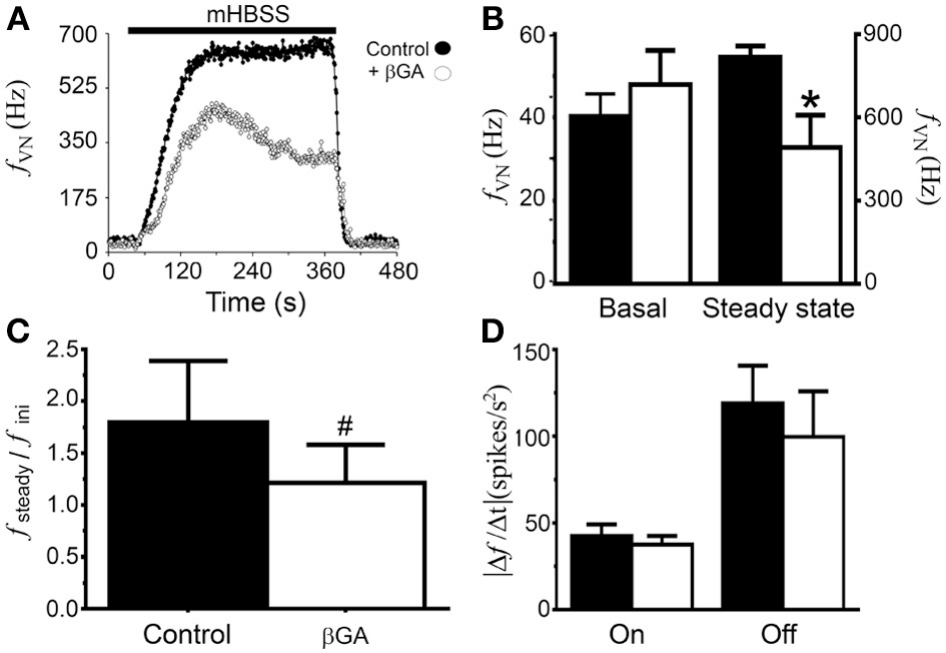
**The enhanced neuronal activity induced by mHBSS is sensitive to βGA**. **(A)** Rat NPJcs were placed in a recording chamber superfused with Hanks' solution (HBSS), followed by superfusion with mHBSS alone (filled circles) or supplemented with 70 μM βGA (empty circles). Bar over the response indicates superfusion with mHBSS. **(B)** Graph showing the average of the basal (pre-stimulus) and steady-state (last 30 s of the response) frequency of discharge in the absence (filled bar) or presence (empty bar) of βGA in the superfusion medium (*n* = 6). **(C)** Graph showing the ratio between the mean steady-state frequency of discharge and mean initial frequency (2 min average after 1 min of the beginning of the stimulation) for each condition. **(D)** Calculated absolute values for the increase and decrease rates of the discharge frequency in the absence (filled bar) or presence (empty bar) of βGA in the superfusion solution. ON represents the rise in frequency when the NPJc is superfused with mHBSS and OFF is the decrease in the frequency of discharge when the ganglion is re-superfused with HBSS. Statistical significances; *P* < 0.05 with respect to control (mHBSS) condition. ^#^Wilcoxon matched-pairs signed rank test; ^*^Student's paired *t*-test. Deviation marks: SE.

Here, we tested the effect of Cx hemichannel blockers (βGA and Gap27) on the neuronal activity of rat NPJc exposed to mHBSS. As mentioned above, when NPJcs were exposed to mHBSS, a rapid and sustained increase in the *f_VN_* was observed, which was maintained for 5 min (Figure 5A, filled circles). However, when NPJc were superfused with mHBSS plus βGA (70 μM), an initial increase in the *f_VN_* was observed followed by a progressive reduction in the maximal frequency until reaching a steady-state. Both, the maximal *f_VN_* and *f_VN_* in steady-state were lower compared with values under control conditions (Figure 5A). Analyses of these results showed no differences between the basal *f_VN_* under control conditions compared with those values before βGA (Figure 5B; *P* > 0.05). However, the steady-state frequency of discharge was reduced from 815.5 ± 42.6 Hz under control conditions to 491.6 ± 116.7 Hz during βGA (Figure 5B, *P* < 0.05). Additionally, the ratio between the steady-state and the maximal initial *f_VN_* was significantly higher in NPJcs under control conditions compared with those values with βGA (Figure 5C; 1.79 ± 0.59 before vs. 1.22 ± 0.37 during βGA; *P* < 0.05). Then, we analyzed the absolute increase rate of the *f_VN_* when HBSS was changed to mHBSS and vice versa. In this case, no difference was found before (42.5 ± 6.7 spikes/s^2^) and during superfusion with βGA (37.6 ± 5.0 spikes/s^2^) (Figure 5D). Similar results were observed in the decrease rate of the *f_VN_* when HBSS superfusion was restored (before 118.8 ± 21.9, v/s during βGA 99.7 ± 26.2 spikes/s^2^) (Figure 5D). These results reveal that Cx hemichannels are partly responsible for the increase in the *f_VN_* when superfused with mHBSS. To test the role of Cx43 hemichannels in this system, we used a Cx43 mimetic peptide (Gap27) (Evans and Leybaert, 2007; Retamal et al., 2007). In mHBSS stop-flow experiments, a final concentration of 50 μM Gap27 was used, and this application reduced neuronal activity by 86.9 ± 6.6%. Similar to that observed with mHBSS, the TATCx43CT effect was also sensitive to Cx- hemichannel blockers. For instance, βGA (70 μM) reduced the TATCx43CT effect by 75.9 ± 14.8% (*n* = 6) and the Gap27 effect by 70.9 ± 7.4% (*n* = 4). Therefore, at least Cxs form functional hemichannels in sensory ganglia cells, and their opening modulates the electrical properties of sensory neurons.

### NPJcs from Panx1 knock-out mice are less sensitive to mHBSS

**Figure 6:**
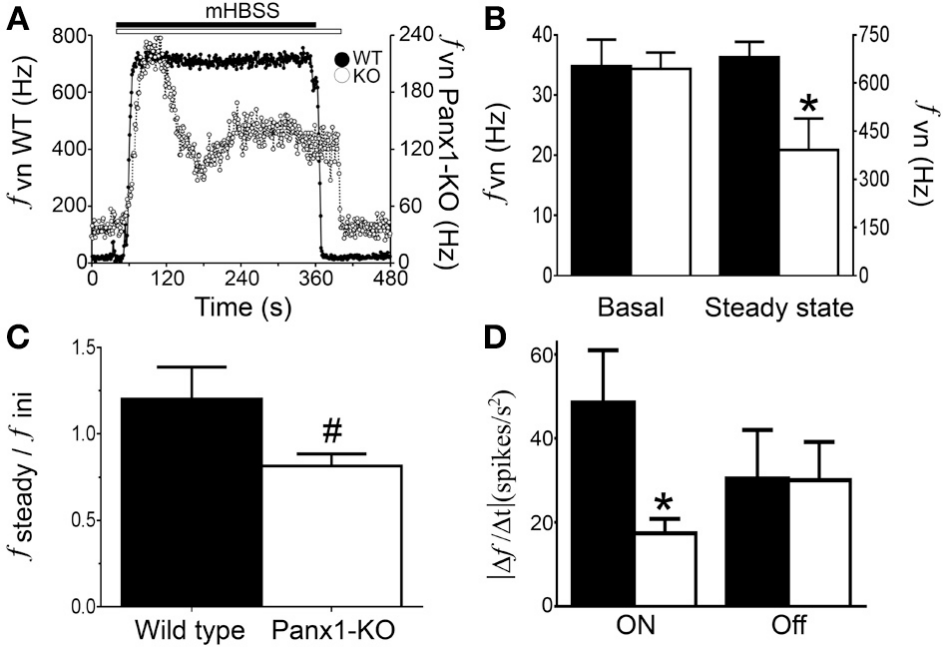
**NPJcs from Panx1 knock-out mice are less responsive to mHBSS**. NPJcs were obtained from wild type (WT) and Panx1 knock-out (Panx1-KO) mice and the mHBSS effect on the vagus nerve activity was tested. **(A)** Representative records showing changes in *f_VN_* induced by mHBSS in NPJcs of WT (filled circles; left Y-axis) and Panx1-KO (empty circles; right Y-axis) mice. Bars on top of the recordings indicate the duration of each stimulus. **(B)** *f_VN_* of NPJcs of WT (filled bar, *n* = 8) and Panx1-KO mice (empty bar, *n* = 9) recorded when the NPJcs were superfused with HBSS (Basal; left Y-axis) or mHBSS (Steady state; right Y-axis). **(C)** Graph showing the ratio between the *f_VN_* in steady state divided by the maximal *f_VN_* reached after mHBSS superfusion (see Figure 3 for definition). **(D)** Graph showing the rate (in absolute value) of the initial changes induced by mHBSS (ON) or the rate of changes when mHBSS was changed to HBSS (OFF) in NPJcs of WT (filled bar, *n* = 8) and Panx1-KO mice (empty bar, *n* = 9). Statistical significance; *P* < 0.05 with respect to WT. ^#^Because these data are unrelated and not normally distributed, we used the Mann-Whitney test, ^*^Student's *t*-test.

Our data suggest that increased neuronal activity induced by mHBSS may also depend on Panx1 channel opening. Therefore, we tested whether sensory neurons from Panx1 Knock out (Panx1-KO) mice could be less sensitive to mHBSS. We found that WT and Panx1-KO mice of similar age and weight presented no obvious morphological differences in their NPJcs (not shown). When HBSS was changed to mHBSS, an abrupt increase in the *f_VN_* was observed in both WT and Panx1-KO NPJcs (Figure 6A). However, in NPJcs of Panx1-KO mice the *f_VN_* subsequently decreased until reaching a new steady-state (Figure 6A, empty circles). Analyses of these data revealed that the basal *f_VN_* values from WT (34.8 ± 4.4 Hz) and Panx1-KO (34.4 ± 2.7 Hz) mice under control conditions did not show significant differences (Figure 6B; *P* > 0.05). However, the steady-state of the *f_VN_* reached under constant mHBSS superfusion showed that the WT activity was ~60% larger than that recorded in NPJcs from Panx1-KO mice (680.6 ± 47.8 Hz, WT vs. 391.6 ± 98.1 Hz, Panx1-KO; *P* < 0.05, Student's *t*-test; Figure 6B). The ratio between the steady state and maximal initial frequency of discharge was significantly lower in NPJcs from Panx1-KO mice, compared with that of WT mice (Figure 6C; 1.20 ± 0.19 WT vs. 0.81 ± 0.07 KO; *P* < 0.05, Mann-Whitney test). Interestingly, the rate of the initial *f_VN_* increase in NPJcs of Panx1-KO animals (17.4 ± 3.5 spikes/s^2^) was slower than that observed in NPJcs of WT mice (48.6 ± 12.5 Hz/s; *P* < 0.05, Student's *t*-test; Figure 6D). Nevertheless, the rate of decrease (when mHBSS was changed to HBSS) of *f_VN_* was similar, as demonstrated when comparing recordings obtained in NPJc of WT and Panx1-KO mice (Figure 6D; *P* > 0.05, Student's *t*-test).

### The activation of the P2X_7_ receptor-Panx1 channel pathway modulates the sensory neuron response to mHBSS

**Figure 7:**
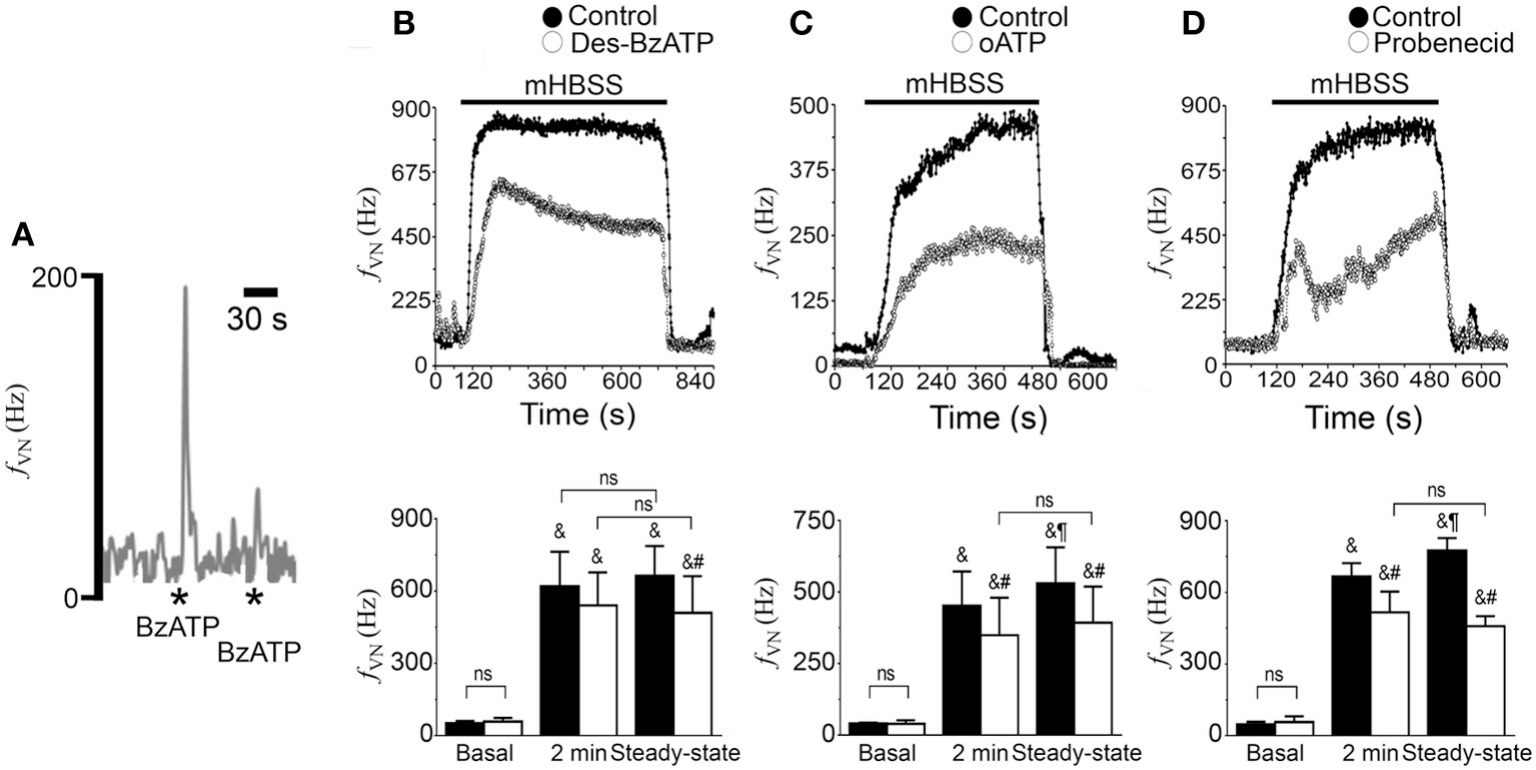
**Neuronal activation induced by mHBSS is sensitive to P2X_7_ receptor-Panx1 channel blockers and neurotransmitters**. Representative recordings of rat NPJcs superfused with mHBSS in the absence (control) or presence of P2X7/Panx blockers. **(A)** 200 μM of BzATP induced a rapid and transient increase in *f_VN_*; however, a second application of BzATP induced only a small response (desensitization). Asterisks show the moment of application of a BzATP bolus to the bath solution. Neuronal frequency of discharge recorded from NPJcs superfused with mHBSS before (Control, filled circles) and after (empty circles) P2X_7_ desensitization (**B**; *n* = 4). Neuronal frequency of discharge recorded from NPJcs superfused with mHBSS alone (Control, filled circles) or supplemented with 100 μM oATP (**C**, empty circles; *n* = 4) with 1 mM probenecid (**D**, empty circles; *n* = 5). (**B–D**; Lower panels). Quantification of the *f_VN_* before superfusion with mHBSS (basal), during 2 min after 1 min in mHBSS and when the *f_VN_* reached a steady state (30 s before the end of mHBSS superfusion) for each corresponding treatment. ^&^Significantly different from basal in the same condition; Newman-Keuls multiple comparisons test. ^#^Significantly different from control conditions (mHBSS), Bonferroni's multiple comparisons test. ^¶^Significantly different from 1 to 3 min (2 min) activity in the same condition. Newman-Keuls multiple comparisons test. Repeated measures 2-Way ANOVA.

It is known that Panx1 channels can be open after P2X_7_ receptor activation (Iglesias et al., 2008; Gulbransen et al., 2012). We used BzATP, which is a P2X_7_ receptor agonist to determine whether the activation of P2X_7_ receptors could mimic the mHBSS effect. We found that a single application of BzATP (200 μM) induced a large (~200 Hz), but brief, increase in *f_VN_* (Figure 7A). However, we noted that a second application of BzATP induced a small response compared with the first response (Figure 7A). This finding indicates that the P2X_7_ receptor in the rat NPJc is desensitized for at least this period of time. Because P2X_7_ receptors can be desensitized by repetitive applications of BzATP, we blocked these receptors. After P2X_7_ receptors were desensitized by repetitive applications of BzATP (200 μM), NPJc were immediately superfused with mHBSS. An increase in *f_VN_* was observed; however, this increase was always smaller than that observed under control conditions. After the initial *f_VN_* increase, the neuronal activity reached a lower steady-state (Figure 7B, *n* = 4). Notably, these responses were similar to those observed in NPJcs of Panx1-KO mice superfused with mHBSS (Figure 6A, empty circles). This finding suggests that the coupled P2X_7_ receptor-Panx1 channel is responsible for maintaining the high level of neuronal activity in the absence of divalent cations. To confirm that P2X_7_ receptors are involved in this phenomenon, we superfused NPJcs with mHBSS supplemented with oATP (100 μM), which is a P2X_7_ receptor antagonist. We found that oATP drastically reduced the maximal frequency of discharge of the vagus nerve in response to mHBSS compared with to control conditions (Figure 7C, *n* = 3). This result is similar to that obtained when P2X_7_ receptors were desensitized. Finally, we tested the effect of probenecid (1 mM), a Panx1 channel blocker. When NPJcs were superfused with mHBSS with probenecid, a reduction in neuronal activity (Figure 7D, empty circles, *n* = 4) compared with control conditions was evident (Figure 7D, filled circles). All of these data support the idea that the Cx hemichannel-P2X_7_ receptor-Panx1 channel pathway is present in the NPJc and modulates the activity of sensory neurons.

### Neurotransmitters modulate sensory neuron responses to mHBSS

**Figure 8:**
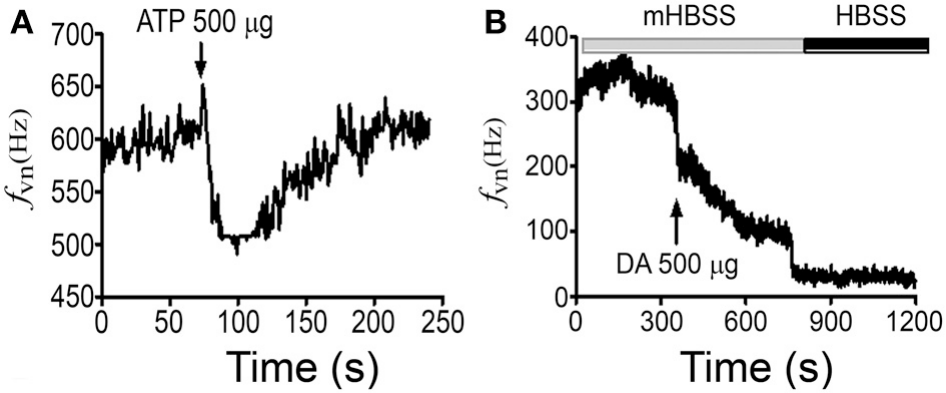
**Neuronal activation induced by mHBSS is sensitive to neurotransmitters**. Representative recordings of electrical activity of rat NPJcs superfused with mHBSS and during steady state activity reached after application of 10 μL of **(A)** 500 μg ATP (*n* = 5) or **(B)** 500 μg dopamine (DA) (*n* = 5).

Sensory neurons express ATP and dopamine receptors (Burnstock, 2009b; Peiser et al., 2009) that upon activation modulate the electrical activity of visceral sensory neurons (Alcayaga et al., 2003). Additionally, these two neurotransmitters are known to modulate channels formed by Panxs and Cxs (Kothmann et al., 2009; Qiu and Dahl, 2009). Therefore, we studied whether ATP and dopamine mediate the enhanced neuronal activity induced by mHBSS. In NPJcs under constant superfusion with mHBSS, boluses of either ATP (10 μl of 500 μg) or dopamine (10 μl of 500 μg) were applied. ATP induces fast and transient decreases in maximal *f_VN_* (Figure 8A, *n* = 5), however, such inhibition was variable between preparations. Similarly, dopamine decreased the maximal *f_VN_*, abolishing the increase in *f_VN_* in 3 of 5 tests. (Figure 8B, *n* = 5).

## Discussion

In this work, we demonstrated that neurons from rat and mouse nodose ganglia present a dramatic increase in activity upon exposure to a Ca^2+^/Mg^2+^-free solution (mHBSS). This enhanced neuronal activity was mimicked to some extent by a peptide that favors Cx43 hemichannel opening and was partially inhibited by Cx hemichannel and Panx channel blockers. According to these results, NPJcs from Panx1 KO mice showed a reduced response to mHBSS, which was similar to the response observed when P2X_7_ receptors were pharmacologically blocked. Therefore, P2X_7_ receptors, Panx1 channels, and Cx43 hemichannels play a role in the enhanced neuronal activity induced by mHBSS, and this activity can be modulated by neurotransmitters, such as ATP and dopamine.

It is accepted that low extracellular Ca^2+^ concentration increases neuronal excitability by direct changes in electrical properties of the neuronal plasma membrane (Frankenhaeuser and Hodgkin, 1957; Hille, 1968). However, in this study, we show that the mHBSS-triggered increase in sensory neuronal activity is partially explained by the activation of Cx hemichannels, Panx channels and P2X_7_ receptors. In support of this notion we found that: (i) this response was sensitive to Cx hemichannel and Panx channel blockers, (ii) the activity of sensory neurons is increased by mHBSS, (iii) P2X_7_ receptor desensitization and oATP partially reduced the increased neuronal activity induced by mHBSS, and (iv) NPJcs of Panx1-KO mice showed a reduced response to mHBSS. Therefore, these results strongly support the notion that the activity of channels formed by Cxs and Panxs does modulate the neuronal activity of the nodose ganglion. Interestingly, the exogenous application of ATP decreased neuronal activity in NPJcs superfused with mHBSS. Accordingly, ATP has been shown to exert a negative feedback control over Panx1 channels, inducing their closure (Qiu and Dahl, 2009). An alternative explanation can be that ATP activates P2X receptors, which in turn overexcites neurons inducing a transient inactivation of voltage-gated Na^+^ channels, which will decrease the number of action potentials, decreasing the *f_VN_*. The inhibitory effect of ATP under mHBSS can be favored by the fact that Mg^2+^ decreases ATP-mediated currents in nodose neurons (Li et al., 1997). Therefore, when Mg^2+^ is removed, ATP can have a greater chance to overexcite sensory neurons, decreasing their activity.

It has been shown that TATCx43CT can restore Cx43 hemichannel activity blocked by high intracellular Ca^2+^ concentrations, thrombin or TATCx43L2 in mammalian cells (Ponsaerts et al., 2010, 2012). Cx43 hemichannels present in sensory ganglia cells appear to be preferentially closed under resting conditions, whereas TATCx43CT releases these hemichannels from this closed state, at physiological Ca^2+^ concentration. In our experiments, the effect of mHBSS on increasing neuronal activity was much more potent than that of TATCx43CT. This result can be due to a combination of direct effects of low extracellular Ca^2+^ concentration on membrane properties and the contribution of other types of Cxs in addition to Cx43. In support of the above hypothesis, we detected Cx26, Cx37 and Cx45 mRNAs in NPJcs. Moreover, it has been reported that injections of capsaicin into a temporomandibular joint transiently increase Cx36 and Cx40 levels in sensory neurons, and Cx26 levels in SGCs of the trigeminal ganglion (Garrett and Durham, 2008). Therefore, the expression of more than one type of Cxs in SGCs and/or sensory neurons of NPJc cannot be ruled out. Thus, we strongly suggest that more than one type of Cx hemichannel is involved in the mHBSS-induced enhanced neuronal activity. Nevertheless, Cx43 appears to have a prominent role because the mHBSS-induced enhanced neuronal activity was drastically inhibited by Gap27, which is a well-known Cx43 hemichannel blocker (Piao et al., 2011; Wang et al., 2012).

Western blot analyses showed several bands of Panx1 that would most likely correspond to glycosylated and non-glycosylated forms because N-glycosylation of Panx1 can change its apparent molecular weight (Penuela et al., 2009). We believe that the non-glycosylated Panx1 is in the cytoplasm and that the glycosylated form forms channels at the plasma membrane of sensory neurons. Consistent with this interpretation, it has been shown that non-glycosylated Panx1 is not delivered to cell membrane and glycosylated Panx1 is observed in the cell surface (Boassa et al., 2007). However, a biochemical study is required to test this hypothesis.

It has been suggested that SGCs and neurons of sensory ganglia present bidirectional communication (Suadicani et al., 2009; Pannese, 2010). Chemical “crosstalk” of sensory neurons has been shown in dorsal root ganglia (Amir and Devor, 1996) and in nodose ganglia (Oh and Weinreich, 2002). However, as mentioned earlier, the somata of sensory neurons are capped by SGCs. Thus, when the somata of sensory neurons release neurotransmitters, such as serotonin, ATP, substance P or acetylcholine (Fueri et al., 1984; Palouzier-Paulignan et al., 1992; Matsuka et al., 2001; Zhang et al., 2007), we propose that these transmitters can reach neighboring SGCs, generating neuronal-glia communication (Suadicani et al., 2009). It is also possible that SGCs release gliotransmitters, such as ATP, that may reach sensory neurons. SGC activation increases intracellular free Ca^2+^ concentration in neighboring neurons via a suramin-sensitive pathway (Suadicani et al., 2009). Therefore, Cx hemichannels and Panx channels could serve as pathways for releasing neuroactive molecules (e.g., ATP and/or glutamate) directly involved in communication between SGCs and neurons. An alternative hypothesis is that sensory neurons and SGCs are connected by gap junction. However, there is no evidence of gap junction plaques between sensory neurons and SGCs and also there is no dye transfer between these cells *in vivo*. However, the above evidence does not exclude the possibility of electrical coupling mediated by small gap junctions between neurons and glial cells that are difficult to see by electron microscopy or to detect with a negatively charged permeability probe such as Lucifer yellow.

The initial increase in *f_VN_* induced by mHBSS was only partially affected by Cx hemichannels and Panx channel inhibitors. However, the maximal *f_VN_* and its maintenance were drastically modified. This result could suggest that these processes are dependent of neurotransmitters released through Cx- and/or Panx-based channels. Because the P2X_7_ receptor is involved, we speculate that at least ATP is important in this process. This hypothesis is consistent with the knowledge that ATP-mediated communication between SGCs and neurons is extremely important (Gu et al., 2010). However, P2X_7_ receptors have been found only in SGCs (Gu et al., 2010). We also observed that sensory neurons express Panx1. Thus, the couple P2X_7_ receptor-Panx1 channel can only be possible if neurons express low levels of P2X_7_ receptor not detected by Gu et al. (2010) and if SGCs present low levels of Panx1 that were not detected in the present work. Single cell mRNA analyses will be required to clarify this point.

In the CNS, when a spinal cord injury occurs, it may induce chronic neuropathic pain. Cx43 expressed in astrocytes play a relevant role in this phenomenon. It has been shown that carbenoxolone (an inhibitor of Cx- and Panx1-based channels) significantly reduces oxaliplatin-induced astrogliosis and mechanical hypersensitivity (Yoon et al., 2013). Additionally, neuropathic pain evoked by weight-drop induces astrogliosis, heat hyperalgesia and mechanical allodynia in WT mice after 4-week post-treatment (Chen et al., 2012). However, these responses almost completely disappeared when Cx43 was deleted (Chen et al., 2012), suggesting that hemichannels and/or gap junction channels formed by Cx43 are critical factors for the development of neuropathic pain. Similarly, SGCs in the PNS may play an important role in the genesis and maintenance of chronic pain (Jasmin et al., 2010; Villa et al., 2010; Hanani, 2012). Additionally, the cascade leading to chronic pain seems to be Cx-dependent (Vit et al., 2006; Ohara et al., 2008; Hanani, 2012). For example, the injection of a GJC and hemichannel blocker prevents the inflammation-induced decrease in the pain threshold in rats injected with Freund's adjuvant (Dublin and Hanani, 2007). Interestingly, SGCs show a reduction in membrane resistance after axotomy of trigeminal neurons (Cherkas et al., 2004), suggesting that some ion channels (e.g., hemichannels) are activated. In contrast, Cx43 hemichannels have been proposed as molecular targets to reduce the inflammatory response of glial and immune cells (Eltzschig et al., 2006; Retamal et al., 2007). This finding raises a question concerning how hemichannels could play a role in pain processing in the PNS. A possible answer is that astrocytes of the CNS control several neuronal functions through ATP, glutamate and NAD^+^ released via Cx43 and Panx1 based-channels (Parpura et al., 1994; Parpura and Haydon, 2000; Higashida et al., 2001; Newman, 2003; Bao et al., 2004). However, the permeability of Panx1 channels to ATP remains a matter of controversy, mainly because Panx1 channels seem to be impermeable to ATP and are blocked by high extracellular ATP concentrations (Qiu and Dahl, 2009; Romanov et al., 2012a,b). Similar to astrocytes of the CNS, SGCs could modulate the activity of sensory neurons through the release of neuroactive substances that may modify the activity of neighboring neurons through Cx43 hemichannels that are permeable to ATP (Stout et al., 2002; Kang et al., 2008), which is an important neurotransmitter related to pain (Burnstock, 2009a). In this study, we observed that mHBSS increased neuronal activity *in vitro* in a Cx43- and Panx1-dependent manner, suggesting that SGCs may release neuroactive molecules that increase the neuronal activity through these channels.

To our knowledge, this work is the first to suggest that P2X_7_ receptors, Cx43 hemichannels, and Panx1 channels may serve as communication pathways between SGCs and sensory neurons somata projecting their axons through the vagus nerve. However, further studies concerning the localization and function of Cx43 and Panx1 in the SGCs are required to understand the physiological and/or pathophysiological role of these pathways in sensory ganglia.

## Author contributions

Mauricio A. Retamal, Julio Alcayaga, and Juan C. Sáez designed research; Mauricio A. Retamal, Julio Alcayaga, Pablo J. Sáez, Christian A. Verdugo, Ricardo Fernández, and Luis E. León performed research; Mauricio A. Retamal, Julio Alcayaga, Christian A. Verdugo, and Juan C. Sáez analyzed data; and Mauricio A. Retamal, Julio Alcayaga, Geert Bultynck, Luc Leybaert, and Juan C. Sáez wrote the paper.

### Conflict of interest statement

The authors declare that the research was conducted in the absence of any commercial or financial relationships that could be construed as a potential conflict of interest.

## Abbreviations

NPJcs: nodose-petrosal-jugular complexes
Cx: connexin (e.g., Cx43)
Panx: pannexin (e.g., Panx1)
SGCs: satellite glial cells
GJC: gap junction cannel
βGA: 18β-glycyrrhetinic acid
HBSS: Hanks' balanced salt solution
GS: glutamine synthetase
BzATP: 2′(3′)-O-(4-benzoylbenzoyl) adenosine 5′-triphosphate triethylammonium
oATP: periodate oxidized adenosine 5′-triphosphate;
OCT: Optimal cutting temperature compound.
